# Nature Studies

**Published:** 1909-12-04

**Authors:** 


### The Practitioner's Relaxations.

### II.—NATURE STUDIES.

BY A MAJOR R.A.M.C.

Larks in Autumn—Bird Songs in the Fall—Badgers—Badgers, Foxes and Rabbits—The Badger in Medicine.

This week was here marked by a heavy gale from the south-west that, for a couple of hours, sent heavy rain swishing down on to us in gusty squalls. A neighbouring town suffered more than we, for there .37 inches fell in less than two hours, and all the drains were choked. After the wind had fallen and the rain had ceased there came calm and bright sunshine. I had occasion to ride across cultivated land at the edge of the Down. As I rode the larks were soaring and singing as sweetly as though it were spring. I saw a pair, the one chivying the other as if with amorous intent, just as they do in spring time. And I saw cock larks sitting on little hummocks, crests erect, looking quite excited. It seemed as if the sudden burst of sunshine and the mildness of the day had made them think of pairing time. Not but what the lark is always an autumn singer. He and the robin are the only birds I hear singing now, excepting that one day a fortnight back I heard a yellow hammer; but he was not singing his full song, "a little bit of bread and no cheese," but a blur only of the first part, the "no cheese" fairly plain.

My landlord came in to see me a day or two ago. He told me that he had found a badger dead on a drive in a wood as he drove through. He could find no marks of violence nor any cause for its death. A queer thing to find a dead badger. I heard of one being found dead two years ago in a gorse cover very sacred to foxes. The badger is fairly common in parts of this county, both in the heavy woodlands and on the open down. I have seen two dug this year, and was present at an unsuccessful attempt to dig another. The attempt failed because of the depth of the pipes and the hardness of a stratum of chalk well impregnated with flint that was struck at the bottom of an eight-foot trench. This particular badger had made his earth in a very large rabbit burrow on a short slope of the open downs. No trees nor bushes near. From the experience of my youth, I knew that the attempt on the old fellow's fort was probably predestined to failure. For the mouths of the pipes he had worked were yards away from one another, and the rabbit burrow very old and well known to have been used by foxes. The terriers—there was a pack of them—were put in singly, and so soon as one came out another was put in. So big was the earth that many of the little dogs failed to find the badger. The presence of rabbits in the earth also drew off all but the experienced.

It is odd that rabbits, badgers, and foxes are so often found together in the same earths. I have known all three kinds in one earth, and often foxes and rabbits, and badgers and rabbits. The rabbits do not seem to mind the presence of their natural enemies. Probably the rabbits keep to the smaller pipes where the larger animals cannot reach them. I believe that the rabbit is the first founder of all the earths in England. I doubt if either fox or badger ever starts his own hole on virgin ground. A rabbit pipe is generally enlarged, and in course of time a vast, irregular network of pipes suitable for a badger grows from it. And these pipes generally, I fancy, follow the old rabbit's ways. I have never seen any sign of a plan in a badger earth.

I wonder how some of the old ideas of the badger originated. Lately I was glancing through an old book of the year 1744—"The Sportsman's Dictionary, or the County Gentleman's Companion"—a queer old book with the *s* like *f*, except at the terminations of words. The author says: "Of this animal there are two kinds, the dog badger so-called on account of its resembling a dog in his feet, and a hog badger, as resembling a hog in his cloven feet." He goes on to describe differences other than that of the feet. He also states that "they live to a great age, and when their sight fails them by reason of old age they keep to their burrows and receive their food from the younger." In fact, the author is full of queer information. He says: "The labour and ingenuity of badgers in making their burrows is worth observation. When they earth, after they have entered a good depth for the clearing of the earth out, one lieth on his back and another layeth earth on his belly; and so taking his hind feet in his mouth draweth him out of the burrow." Truly most worthy of observation if true.

The author speaks also of the medicinal value of the badger thus: "There are the following profits and advantages which accrue by killing this animal. Their flesh, blood, and grease, tho' they are not good for food, yet are very useful for physicians and apothecaries for oils, ointment, salves, and powders for shortness of breath, the cough of the lungs, for the stone, sprained sinews, colt aches, etc., and the skin, being well dressed, is very warm and good for antient people, who are troubled with paralytic distempers." I wonder what a "colt ache" is in modern medicine.

